# Internal hernia through mesoappendix causing small bowel obstruction: A case report

**DOI:** 10.1016/j.ijscr.2024.110590

**Published:** 2024-11-12

**Authors:** Eneyew Mebratu Gashey, Sileshi Genetu Tiruneh, Fekadu Wudie Gelaw, Adugna Tasew Tebabel, Litegebew Yitayeh Gelaw

**Affiliations:** a Department of Surgery, GAMBY Medical and Business College, Bahirdar, Ethiopia; b Department of Pathology, Bahirdar university, Bahirdar, Ethiopia; c GAMBY Medical and Business College, Bahirdar, Ethiopia; d Beza Hiwot Diagnostic Center Bahirdar, Ethiopia

**Keywords:** Case report, Hernia, Small bowel obstruction, Mesoappendix, Laparotomy

## Abstract

**Introduction:**

Intestinal obstruction is one of causes of acute abdomen leading to laparotomy. Even though there are different causes of small bowel obstruction (SBO), internal hernia is a rare one. Though different types of internal hernias occur, herniation through the mesoappendix is reported only four times in the literature.

**Case presentation:**

We describe here such a type of hernia on a 50 yrs old male presented with symptoms and signs of SBO whose cause was diagnosed to be internal hernia through mesoappendix intraoperatively. He was managed with appendectomy and release of the hernia.

**Clinical discussion:**

Intestinal obstruction is a common cause of acute abdominal conditions and can lead to significant mortality if not managed appropriately. Internal hernias account for less than 1 % of intestinal obstruction cases. The cause of trans-mesoappendicular internal hernias remains largely unknown. A congenital defect in the mesoappendix may contribute to the development of these hernias.

**Conclusion:**

In patients with acute intestinal obstruction without previous surgery, internal hernia should be considered as a differential diagnosis. Trans-mesoappendicular hernia is extremely rare type of internal hernia.

## Highlights

• Internal hernia is herniation of a part of bowel through congenital or acquired openings within abdominal cavity.
• Trans-mesoappendicular hernia is a rare internal hernia where herniation occurs through an opening on the mesoappendix.
• Clinically patients usually present with abdominal pain, vomiting and constipation.
• Immediate diagnosis and management saves life

## Abbreviations

SBO: Small bowel obstruction
IAH: Internal abdominal hernia
CT: Computed tomography
REEA: Resection and end-to-end anastomosis

## 1 Introduction

An internal abdominal hernia (IAH) refers to the protrusion of abdominal viscera, most commonly small bowel loops, through a peritoneal or mesenteric defect into the abdominal or pelvic cavity [1]. Internal hernias are rare causes of intestinal obstruction, with an incidence rate of 0.2 % to 0.9 % [2]. In developed nations, intra-abdominal adhesions are the primary cause of small bowel obstruction (SBO), responsible for approximately 74 % of cases [3]. Hernias and volvulus are the common reasons for intestinal obstruction in developing nations, volvulus being the commonest cause in Ethiopia [4].

More than half of internal hernias are located paraduodenally, while other identified types include transmesenteric, supravesical, perivesical, intersigmoid, foramen of Winslow hernias, and omental hernias [5]. Patients with congenital defects often present symptoms in childhood. One particularly rare internal hernia occurs through the mesoappendix. To our knowledge, there are only four documented cases in the literature to date [[6], [7], [8],^12^].

In this report we presented a case of small bowel obstruction due to internal hernia through mesoappendix.

This work has been reported in line with the SCARE criteria [9].

## 2 Case presentation

A 50-year-old male patient presented to our hospital with a complaint of severe crampy abdominal pain of two days duration. He reported frequent episodes of bilious vomiting but denied any history of constipation. The patient had no previous abdominal surgeries and no known medical conditions.

On physical examination, the patient was acutely ill, in pain. His pulse rate was 92 beats per minute, while other vital signs remained within the normal limits. Abdominal examination revealed mild distension with visible peristalsis. There was direct and rebound tenderness over the abdomen particularly in the right lower quadrant and suprapubic area. No surgical scars were observed. Inguinal areas were free.

**Fig. 1:**
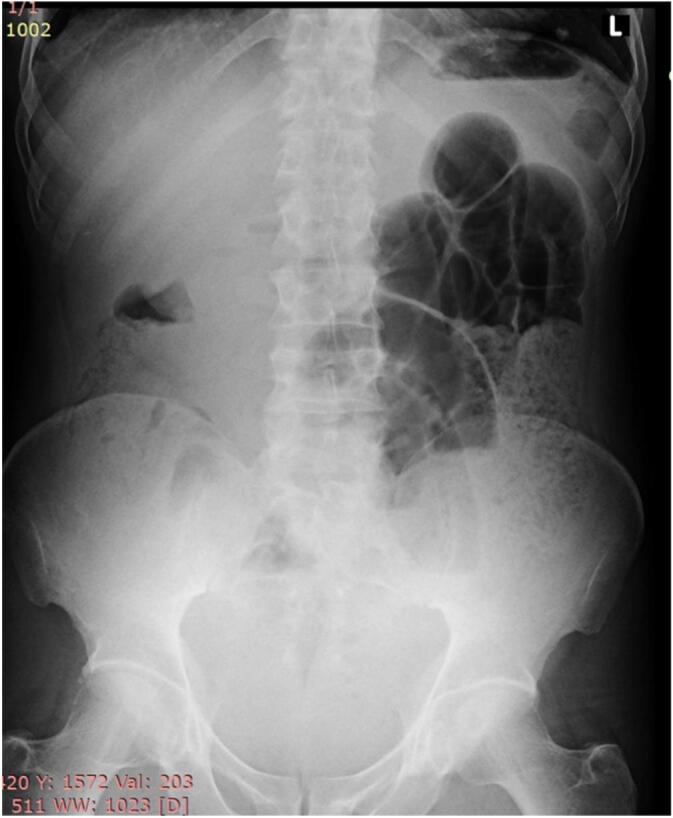
Erect plain abdominal film showing signs of small bowel obstruction.

Basic blood workups were within normal limits. Abdominal ultrasound revealed distended small bowel loops with clear free peritoneal fluid. An erect plain abdominal film demonstrated multiple air-fluid levels with distended small bowel loops, suggestive of small bowel obstruction (Fig. 1).

**Fig. 2:**
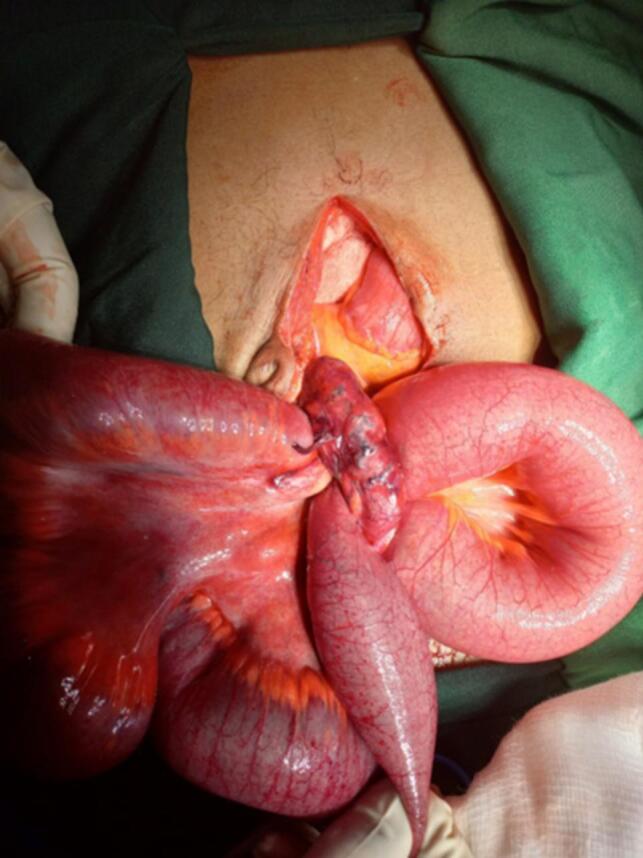
Intraoperative photograph showing herniated bowel through mesoappendix.

**Fig. 3:**
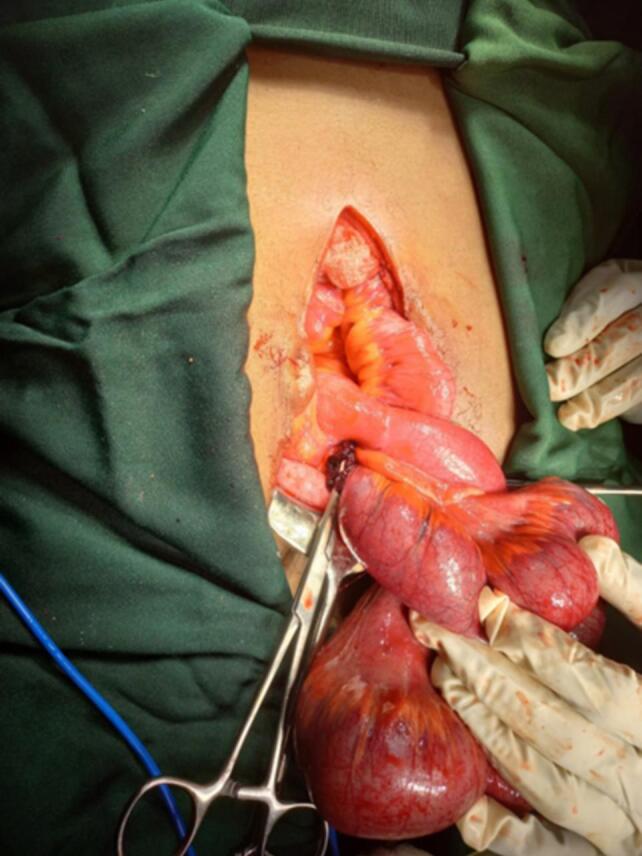
Intraoperative photograph after transection of meso-appendix.

The patient was resuscitated and prepared for an emergency open laparotomy. Under general anesthesia, the abdomen was opened, revealing approximately 200 mL of reactive fluid and distended, dusky small bowel loops. A defect in the mesentery of the appendix allowed a segment of ileum, approximately 50 cm long and 60 cm from the ileocecal valve, to herniate through (Fig. 2, Fig. 3). The mesoappendix was divided, and the entrapped bowel was released, returning to a pink coloration post-decompression. An appendectomy was performed.

The patient's recovery was uneventful, and he was discharged after three days of stay in the hospital, demonstrating significant improvement. At the one-month follow-up, the patient reported no complaints, and his surgical wound had healed well.

## 3 Discussion

Intestinal obstruction is a common cause of acute abdominal conditions and can lead to significant mortality if not managed appropriately [8]. Internal hernias account for less than 1 % of intestinal obstruction cases [2]. An internal hernia is characterized by the protrusion of viscera through either a normal or abnormal opening within the peritoneal cavity [7].

The transmesenteric hernia was first described by Rokitansky in 1836 [11]. While mesenteric defects are observed in approximately 0.5 % of autopsies, cases of transmesenteric hernias in adults remain uncommon [1]. Typically, these defects are located near the ligament of Treitz or the ileocecal valve [6].

Mesenteric defects can be classified as either congenital or acquired. Congenital mesenteric defects are believed to result from prenatal intestinal ischemia, which leads to the thinning of the mesenteric leaves. In contrast, acquired internal hernias often arise following gastrointestinal surgeries, with transmesocolic hernias being the most common type associated with procedures like Roux-en-Y gastric bypass surgery [1].

The cause of trans-mesoappendicular internal hernias remains largely unknown. A congenital defect in the mesoappendix may contribute to the development of these hernias [8]. Mesenteric defects can present with a wide range of clinical symptoms, from being asymptomatic to resulting in unexpected fatalities [10]. The clinical presentation often mimics other causes of small bowel obstruction, making preoperative diagnosis difficult [1,5,8].

Accurate clinical examination and imaging studies are crucial for diagnosis. Computed tomography (CT) is considered the gold standard, with a specificity of 76 % and a sensitivity of only 63 % [1,8]. Urgent surgical intervention is essential to prevent strangulation, a condition associated with high mortality rates. Delays in treatment can lead to severe complications due to gangrenous bowel [1].

Surgical management typically involves a timely laparoscopy or laparotomy, hernia reduction, closure of the defect, and if necessary, resection of any nonviable bowel and in our case doing appendectomy [10].

*Trans*-mesoappendicular hernia is a rare occurrence with only four cases reported on English literatures (Table 1).

**Table 1:** Trans-mesoappendicular hernias reported in English literatures yet.

Year	Age/sex	Author	Presentation	Diagnosis (hernia content)	Management
1963	80/M	Rooney JA [6]	SBO	Ileum	Bowel reduced, defect closed
2016	5/M	Bar man [7]	SBO	Meckel's diverticulum	Content reduced, Resection and end-to-end anastomosis (REEA) of ileum, defect closed
2020	55/M	Vinay HG [8]	SBO	Ileum	Content reduced, appendectomy
2020	33/F	CE Jones [12]	Acute appendicitis	Rt ovary	Ovary reduced, appendectomy (lap)
2024	50/M	Our case	SBO	Ileum	Ileum reduced, appedectomy

## 4 Conclusion

In patients with acute intestinal obstruction without previous surgery, internal hernia should be considered as a differential diagnosis. *Trans*-mesoappendicular hernia is extremely rare type of internal hernia. CT scan can be done pre-operatively in a stable patient, but laparotomy often establishes the diagnosis. Early exploration after initial stabilization will help avoid potential life threatening complications.

## Consent

Written informed consent was obtained from the patient for publication of this case report and accompanying images. A copy of the written consent is available for review by the Editor-in-Chief of this journal on request.

## Ethical approval

Ethical approval for this study was provided by ethical committee of GAMBY Medical and Business College on 5th July 2024 with Ref. No G/C-499/2024.

## Funding

The authors received no financial support for writing or publication of this article.

## Author contribution

All authors contributed to the conception, writing, and editing of the case report.

## Guarantor

Eneyew Mebratu Gashey

## Declaration of competing interest

We declared that there is no conflict of interest.
